# Towards a framework for invasive aquatic plant survey design in Great Lakes coastal areas

**DOI:** 10.3391/mbi.2022.13.1.03

**Published:** 2022-02-04

**Authors:** Andrew J. Tucker, Gust Annis, Erick Elgin, W. Lindsay Chadderton, Joel Hoffman

**Affiliations:** 1 The Nature Conservancy, 721 Flanner Hall, University of Notre Dame, IN 46556, USA; 2 The Nature Conservancy, 101 E. Cesar E. Chavez Ave, Lansing, MI 48906, USA; 3 Michigan State University Extension, 160 Agriculture Hall, East Lansing, MI 48824, USA; 4 USEPA Great Lakes Toxicology and Ecology Division, Duluth, MN, USA

**Keywords:** surveillance, monitoring, early detection, rarefaction, aquatic macrophytes

## Abstract

At least 65 aquatic plant species have been identified as part of a surveillance list of non-native species that pose a threat to biodiversity and ecosystem services in the Laurentian Great Lakes. Early detection of these potentially invasive aquatic plants (IAP) could minimize impacts of novel incursions and facilitate successful eradication. We developed, implemented, and then adaptively refined a probabilistic boat-based sampling design that aimed to maximize the likelihood of detecting novel IAP incursions in large (400+ hectares) Great Lakes coastal areas. Surveys were conducted from 2017 to 2019 at five Great Lakes locations – St Joseph River (MI), Saginaw River (MI), Milwaukee (WI), Cleveland (OH), and the Detroit River (MI). Aquatic plant communities were characterized across the five sites, with a total of 61 aquatic plant species detected. One-fifth of the species detected in our surveys were non-native to the Great Lakes basin. Sample-based species rarefaction curves, constructed from detection data from all surveys combined at each location, show that the estimated sample effort required for high confidence (> 95%) detection of all aquatic plants at a site, including potentially invasive species, varies (< 100 sample units for Detroit River; > 300 sample units for Milwaukee, roughly equivalent to 6 to 18 days sampling effort, respectively). At least 70% of the estimated species pool was detected at each site during initial 3-day surveys. Leveraging information on detection patterns from initial surveys, including depth and species richness strata, improved survey efficiency and completeness at some sites, with detection of at least 80% of the estimated species pool during subsequent surveys. Based on a forest-based classification and regression method, a combination of just five variables explained 70% or more of the variation in observed richness at all sites (depth, fetch, percent littoral, distance to boat ramps and distance to marinas). We discuss how the model outcomes can be used to inform survey design for other Great Lakes coastal areas. The survey design we describe provides a useful template that could be adaptively improved for early detection of IAP in the Great Lakes.

## Introduction

The Laurentian Great Lakes ecosystem is heavily invaded, with over 188 non-native aquatic species considered established, including 61 invasive species for which measurable negative environmental or socio-economic impacts have been documented (Sturtevant et al. 2019). These numerous invaders endanger other aquatic ecosystems because the Great Lakes act as an important beachhead for the spread of aquatic invasive species (AIS) to the inland waters of the basin and across North America (Rothlisberger and Lodge 2013). Consequently, Tribal, Canadian Provincial, and United States Federal and State governments have invested significant resources to manage the AIS threat, with management efforts historically focused on preventing AIS introduction from key pathways, but increasingly directed at the establishment of regional surveillance programs to facilitate early detection and rapid response to novel introductions (International Joint Commission 2017).

Great Lakes regional surveillance programs have been designed either to detect a select few high-profile species (e.g. Asian carps, Jerde et al. 2013; Eurasian Ruffe, Tucker et al. 2016, Bowen and Keppner 2013), or to detect entire communities of fish or invertebrates, primarily at major ports (Hayer et al. 2017). The emphasis on community level fish and invertebrate surveys at Great Lakes ports is largely a result of the historic importance of shipping as the major vector for introduction of AIS to the Great Lakes (Grigorovich et al. 2003; Ricciardi 2006) and also reflects the taxonomic focus of the US Fish and Wildlife Service (FWS), who have led program implementation (USFWS 2014; Harris et al. 2018). As a result, there has been substantial progress towards refining fish, benthic invertebrate, and zooplankton surveillance methods and survey design in the coastal waters of the Great Lakes (Trebitz et al. 2009; Hoffman et al. 2011, 2016).

Comparatively, we know little about the best approach for effectively detecting invasive aquatic plants (IAP) in Great Lakes coastal waters. IAP surveillance was a key management gap identified as part of a regional collaboration focused on developing a framework for early detection monitoring in the Great Lakes (Chadderton et al. 2021). The regional framework identified a list of surveillance species with the greatest potential to arrive, establish, and cause impacts in the Great Lakes. Nearly half (65 of 144) of the species of concern were aquatic plants (Davidson et al. 2021). The locations representing the nexus of pathways most likely to introduce these priority IAP included various ports, estuaries, embayments, and drowned river mouths, many of them relatively large (~ 10 km^2^) and complex, with a diversity of open water habitats (shallow, deep, exposed, sheltered, rocky, sandy, etc.). The locations with highest risk of IAP introduction are spread across the basin but are especially concentrated in southern Lake Michigan and western Lake Erie in areas with high densities of natural or artificial connections, boat launches and marinas, or large population centers (Tucker et al. 2020).

Our aim in this study was to test and refine a survey method for early detection of IAP across the range of Great Lakes ports and open water coastal areas where they are expected to arrive. We had two key objectives: 1) determine the effort required for high-probability early detection of IAP in Great Lakes open water coastal habitats, and 2) compare species detection among different survey designs, to inform progress towards an efficient and comprehensive surveillance approach. In the absence of a tested protocol we adopted a quantitative, probabilistic, and adaptive survey design approach that has been used to develop surveillance protocols for fish and invertebrates in the Great Lakes (e.g. Trebitz et al. 2009; Hoffman et al. 2011). To ensure survey methods were accessible (i.e. not requiring specialist training or equipment) sampling was conducted using methods common to aquatic plant surveys (i.e. visual meander and rake tosses). To decrease the likelihood that some habitats were not sampled at all and since resource constraints preclude systematic sampling of areas as large as an entire coastal embayment or estuary, we randomly selected sample units from across the entire site in initial surveys at each location. We allocated effort proportionally towards species rich habitats in subsequent surveys as we learned where we were likely to find plants. We used species accumulation theory to infer the probability of detecting species given a prescribed level of sampling effort and to evaluate whether more focused survey designs improved survey performance measures. Finally, we used a forest-based classification and regression method to evaluate how well different combinations of spatial data layers (i.e. habitat variables) predicted observed aquatic plant richness measures from our surveys. Our goal was to leverage the classification method to identify key habitat variables that could be used to direct surveys towards habitats most likely to support IAP.

## Materials and methods

### Study area

**Figure 1.**
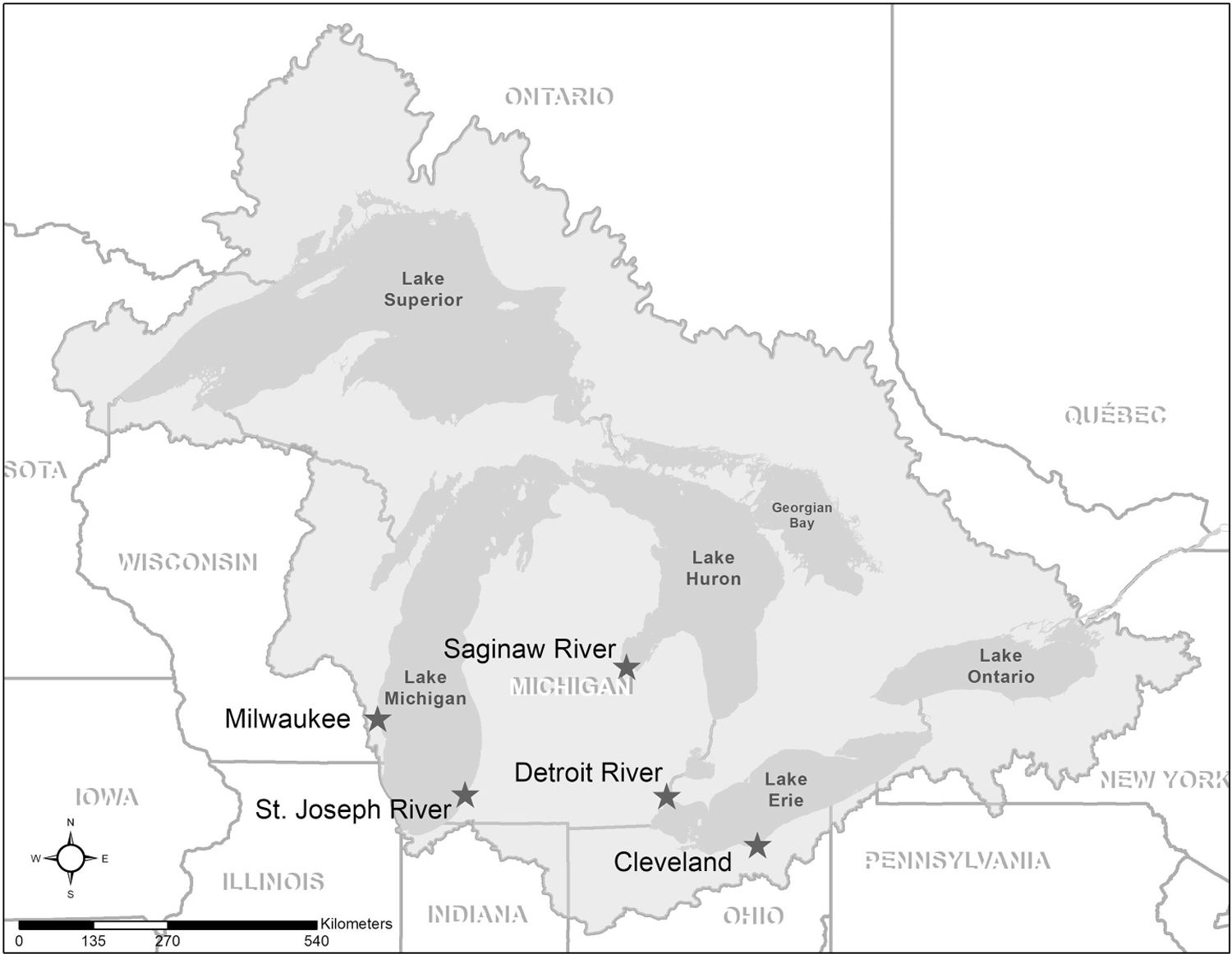
Great Lakes basin showing the five sites where aquatic vegetation sampling occurred. Data credits: Lakes: Great Lakes Aquatic Habitat Framework (GLAHF) Great Lakes shoreline v 1.1, 2014. Basin: Institute for Fisheries Research Great Lakes GIS basin outline GLB_basin_outline_noSLS_IFR, 2004. States/Provinces: ArcGIS Content Team U.S. States and Canada Provinces, 2010.

We selected for sampling five Great Lakes coastal areas where propagule pressure and introduction risk measures for IAP are among the highest in the basin, Cleveland (OH), Milwaukee (WI), St. Joseph River (MI), Saginaw Bay (MI), Detroit River (MI) (Figure 1; Tucker et al. 2020). Sites vary in size (400–800 hectares) but each consists of a mixture of habitat types characteristic of Great Lakes coastal areas and ports, where plant abundance and diversity are expected to vary with local conditions (e.g. depth or transparency, Hutchinson 1975; Middelboe and Markager 1997). Some sites are mostly shallow (< 3 m on average) and transparent with a mix of natural shoreline or engineered shoreline with docks (e.g. Detroit River), while other sites are relatively deep (maximum depth 10 m or more) and turbid, with largely hardened waterfronts and a few large marinas (e.g. Cleveland). The species of concern represent a full range of growth forms and will occur in any number of these habitat types, from emergent grasses along natural shorelines (e.g. Phragmites), to submerged macroalgae in deeper harbors (Starry stonewort), to free-floating lilies adjacent to coastal wetlands (e.g. European frog-bit). We sought to employ a survey design and sampling methods that would effectively sample this diversity of habitats.

### Survey design

Sampling was initiated under an adaptive monitoring framework: a cycle of annual surveillance, evaluation, and improvement. The goal of initial surveillance was to survey all habitats and to acquire supporting environmental or species composition data (e.g., depth or species richness) to evaluate ecological patterns that might facilitate more efficient early detection (Trebitz et al. 2009). In general, for first-time surveys, the site was partitioned into sample zones a priori based on major habitat types (e.g. inner harbor, outer harbor, river). For the St. Joseph River, which was only sampled in 2017, we allocated sampling across three zones that we expected would vary with respect to flow and depth (shallow/high flow upper river, deep/low flow lower river, and shallow/low flow tributary confluence). The sample zones were then manually superimposed on each site in GIS and sample units (100 m per side) were overlaid. A subset of the sample units was randomly selected from each zone using the create spatially balanced points tool in ArcGIS (version 10.5.1). This tool selects sample locations based on an algorithm proposed by Theobald et al. 2007. The key input to the tool is a raster defined by the user that specifies the sample frame and the inclusion probability values for each sample location (between 0 and 1), with higher values more likely to be selected for sampling. We specified at least ten percent (of total sample units in a zone) as a minimum lower bound on the number of samples selected from each zone, though only a subset of the selected locations were ultimately sampled. In Saginaw (which was only sampled in 2018), the survey area was a priori partitioned into shallow (< 4 m) and deep (> 4 m) habitat zones. Sample units were randomly selected using the “create spatially balanced points” tool but we specified that 75% of the sampling effort would be allocated to sites in the shallow zone based on evidence from surveys at other sites in 2017 that suggested higher incidence of plants in shallow areas.

**Figure 2.**
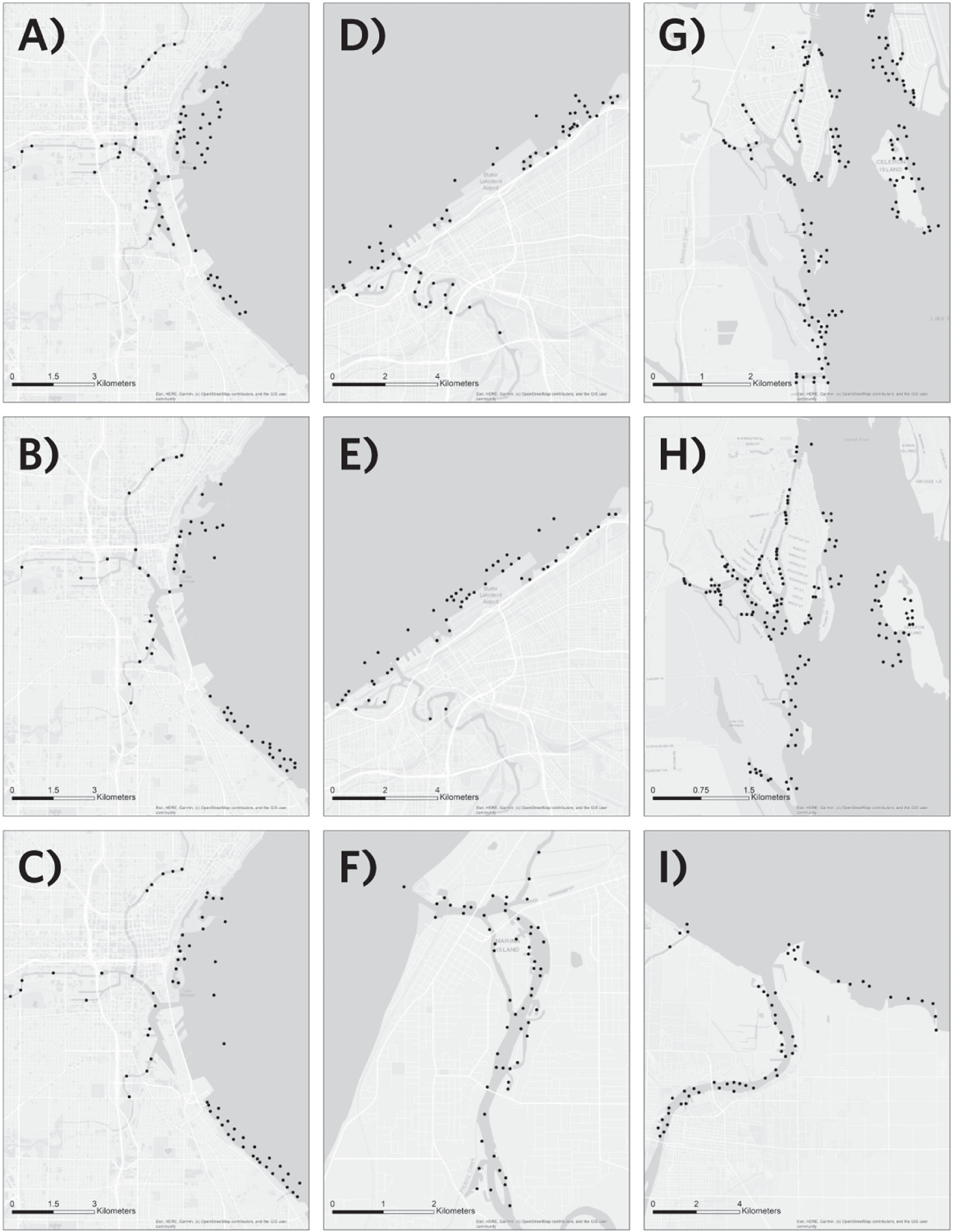
Sample units surveyed by year (points indicate sample unit centroids). Milwaukee, 2017–2019 (A–C), Cleveland 2017, 2019 (D, E), St Joseph River 2017 (F), Detroit River 2018, 2019 (G, H), Saginaw River 2018 (I).

At sites where surveys were conducted more than once (Milwaukee, Cleveland, Detroit River), we maintained the same spatial extent as in initial surveys but allocated samples over a limited space by targeting shallow areas or areas where earlier sampling yielded high species richness (Figure 2). For Milwaukee 2018 (year 2), sample units were randomly selected from shallow (< 4 m) and deep (> 4 m) zones as described above for Saginaw River. For Milwaukee 2019 (year three), we used results from the year one and year two Milwaukee surveys to develop a species richness surface using kriging in ArcGIS. The richness surface was limited to areas less than six meters deep and was overlaid on the grid of sample units (100 m per side). Probability weights based on richness were assigned to each sample unit and the “create spatially balanced points” tool in ArcGIS was used to randomly select 70 sample units across the whole site (i.e. 90^th^ percentile = 0.6, 80^th^ percentile = 0.5, 70^th^ percentile = 0.4, 60^th^ percentile = 0.3, 50^th^ percentile = 0.2, < 50^th^ percentile = 0.1) We used the same approach, based on observed species richness from initial surveys, for year two surveys at Cleveland and Detroit River, except that the richness surface produced for Detroit River was not depth limited (since no portion of the site exceeded 6 m).

### Sampling method

Surveys were conducted by boat in August or September, 2017–2019. Surveys were constrained to three-day visits for logistical reasons (i.e. to balance survey thoroughness with realistic effort). At each sample unit, plants were collected with rakes or observed visually and recorded. The rakes were double headed 14-tine rake heads attached to approximately 50 feet of 3/8” (15.24 meters of 0.95 cm) braided polypropylene rope. The survey targeted submerged, floating, and emergent taxa. All observations were from the shoreline lakeward to the maximum depth in any grid cell. Plants were only recorded if they were observed in water (i.e. plants observed growing above the waterline were not included in the survey). The location of a sample unit was occasionally adjusted away from the a priori random location selected in ArcGIS to sample adjacent areas where field observations indicated presence of macrophyte beds.

In general, four evenly spaced and discrete stations were sampled at each sample unit (e.g. the four corners of the grid), though areas where plants were present were targeted. In deep and/or turbid areas, where river/harbor bottom was not visible from the boat, a transom mounted portable sonar unit (Garmin Striker 4) was used as an aide to identify sampling stations within the sample unit (based on potential presence of macrophytes from sonar imaging). At each station a rake was tossed to a distance 5–10 meters from the boat, allowed to sink to the river/harbor bottom, and slowly retrieved. At all sampling stations the aim was to sample until no new species were detected, but always with a minimum level of effort. Thus, a minimum of four rake tosses occurred at each station (i.e. one toss from each side of the boat). In instances where the fourth rake toss resulted in collection of a species not observed in the previous three tosses, subsequent rake tosses were made until no new species were collected. The sampling area (up to 10 m in all directions from the boat) was also searched visually and species were collected as needed for identification (sensu Yin et al. 2000).

Plants were identified to species in the field following Crow and Hellquist (2000a, b). When specimens could not be identified in the field (e.g. when identification required scrutiny of morphological features under a microscope) the plant was retained and identified to species (or lowest possible taxonomic group) in the laboratory. It was not feasible to identify narrow-leaf *Potamogeton* spp. (leaf width < 2 mm) to species in the field due to the pace of the protocol. Representative narrow-leaf *Potamogeton* specimens found in various survey locations were identified to species after the fact in the laboratory. Certain genera, including *Sparganium* and *Sagittaria* were only identified to species level when flowers or fruits were present. Plants in the genus *Wolffia* were not resolved to species level. Non-angiosperms, including *Chara* and *Nitella* were identified to genus only, except for starry stonewort (*Nitellopsis obtusa*). Freshwater mosses were identified as *Drepanocladus* spp. We did not identify *Typha* to species because using morphological traits to distinguish between *Typha latifolia*, *T. angustifolia* and *T.* x *glauca* can be misleading (Geddes et al. 2021). A representative specimen of each taxa collected was vouchered and deposited with a relevant state herbarium. The total number of species collected at each sample location from across all rake tosses was tallied for data analysis. Water depth at each sample station was recorded from the portable sonar unit. GPS coordinates, plant species observed, and water depth at each sample station were recorded electronically in the field to an iPad (connected to a Bluetooth enabled GPS receiver) using Collector for ArcGIS. Secchi depth was measured at a subset of sampling locations during each site visit.

### Analytical methods

For analysis, the species richness value for each sample unit was the aggregate from the four stations in each sample unit. Data were analyzed after Hoffman et al. (2011). Species accumulation theory was used to estimate species richness and characterize species accumulation for each survey, as well as to quantify the effort required for high-probability rare species detection at each site (a proxy for difficult-to-encounter non-native species). We generated species accumulation curves using the sample-based rarefaction curve. We report the 95% confidence intervals (CIs), as well as an estimate of the lowest possible percentage of the estimated total species richness that could be calculated for the first one or few samples (based on the rarefaction curve, assuming random reordering of samples), as a proxy for the initial species accumulation rate. The faster the rate at which the curve rises, the more species-rich the data set will be (i.e., there are more species per individuals encountered).

To estimate total species richness (Sest) we used a nonparametric, sample-based estimator and calculated the Sest values (“Chao2”) using the EstimateS v8.20 software (Colwell 2006). Following Colwell (2006), we report bias-corrected Sest values unless there was substantial heterogeneity among species in their probabilities of detection (incidence-based coverage estimator of species richness coefficient of variation (CV) > 0.5), in which case we report the uncorrected Sest value. To quantify the effort required for high-probability nonnative species detection, we estimated the number of samples required to obtain a near-total (95%) or total (100%) census of the plant species present. We used the nonparametric method proposed by Chao et al. (2009), which calculates the number of samples required to detect some proportion of the asymptotic sample-based species richness estimator Sest. We examined species overlap among designs using the Simplified Morisita index, which measures the similarity of species’ incidence with values ranging from 0 (no shared species) to 1 (complete overlap). Mean values for rare, non-native, and total species richness were also calculated. Species classified as “nonindigenous” per the Great Lakes Aquatic Nonindigenous Species Information System (GLANSIS) were counted as non-native. Rare species were defined as native or non-native species comprising < 5% of the total incidence at a site. Total richness was the sum of all native and non-native species. The relationship between observed richness of native species versus non-native species was analyzed graphically.

We used a forest-based classification and regression approach to predict species richness values at the sample-unit scale for each site (ArcGIS Pro v2.6.3). Model predictions were based on a set of categorical and continuous habitat attributes associated with abiotic conditions that are known to influence plant establishment and surrogates for aquatic plant pathways of introduction, including: fetch, depth, distance to shore, shoreline hardness, distance to boat launches, distance to marinas and distance to docks (Supplementary material Table S1). Measures for each variable were attributed in ArcGIS for each sample unit. Distance measures were determined from the edge of each sampling unit grid cell.

Model predictions for sample-unit species richness were generated for each site based on the algorithm derived from site level species richness data. We report model fit statistics (i.e. r2 values from site specific data that show how well the model predicted observed richness) at each site for three different models: an all variable model (n = 18 variables), a reduced variables model, and a depth only model (using mean depth). The reduced variable model was generated using five variables that we selected based on importance to the all variables model with additional consideration given to the ease with which a user could produce the variable layer for a novel site using commonly available GIS datasets and processes. Our intent was not to find the “best model” per se, but to find a useful model that would accurately predict richness based on a few easily accessible (or easily derived) spatial layers. Variable importance was calculated from Gini coefficients, where a higher coefficient indicates a more meaningful variable. The variables selected for the reduced variable model registered importance values that were ≥ 5% of the total sum of Gini coefficients and included mean depth, percent littoral zone, maximum fetch, minimum distance to boat launch, and minimum distance to marina. We also produced a depth only model to demonstrate model performance using only the single top predictor variable from the all variables model.

## Results

### Survey effort

The number of sample units surveyed at any one site varied between 39 and 73 (Table 1). At least 5% and as much as 19% of the total sample frame was sampled during each survey (i.e. proportion of total sample units that were sampled with rakes). Proportionally more survey effort was allocated to some “zones” than to others within sites. For example, a larger proportion of the inner harbor at Cleveland and Milwaukee was sampled compared to the outer harbor at those sites (18% and 8% versus 5% and 4%, respectively). For sites that were sampled more than once, shallow or species rich areas within each site were sampled more intensively than deep areas or areas with low predicted richness.

### Observed and estimated species richness

Sixty-one species were collected from across the five sites (Table S2). One-fifth of the taxa collected were non-native to the basin (n = 12), including the first detection of *Cabomba caroliniana* at the St. Joseph River site. All other non-native species were previously known from the locations where they were detected. Various growth forms were observed, though the majority (54%) of detections were submerged aquatic plants (Table S3). Observed species richness for any single survey ranged from 16 species (Milwaukee 2017) to 41 species (Saginaw 2018). Estimated richness, from rarefaction, was also lowest at Milwaukee 2017 (23 species) and highest at Saginaw (56 species; Table 2). At all sites, observed non-native and native richness were positively correlated (Figure S1). In a small number of cases, non-native richness was greater than native richness at the sample unit level, but only on the low end of the richness spectrum and by a difference of at most one species (e.g. one non-native species to zero native species, or three non-native species to two native species).

Observed species richness (S_obs_) increased at all sites during year two or year three surveys compared to year one generalized random surveys (Table 2). At Milwaukee, where a total of 34 species were observed across the three years, only 16 species (< 50% of total) were observed in year one compared to 26 species and 25 species in year two and year three surveys, respectively. Species richness estimates from rarefaction curves vary from year to year but converge on more precise estimates of overall richness when survey data from all survey years are combined. For example, the S_est_ for Milwaukee from the year one survey was 34 species (95% CI [17–58]), for year two 31 species (95% CI [27–50]), and for year three 41 species (95% CI [28–108]). When all three survey years are combined, the S_est_ is 40 species (95% CI [35–59]).

Values for all observed richness endpoints (total, rare, and non-native) increased in year two and year three surveys when compared to year one (Table 3; Figure S2). The largest year over year increases were observed at Milwaukee. At Detroit River, rare and non-native richness decreased, but marginally, reflecting the relatively homogenous distribution of plants at that site. The simplified Morisita index revealed a high proportion of shared species among year one and year two designs for Detroit and also for Cleveland, but for Milwaukee the year one survey was more dissimilar to the year two and year three designs than the year two and year three designs were to each other (Table 4).

**Figure 3.**
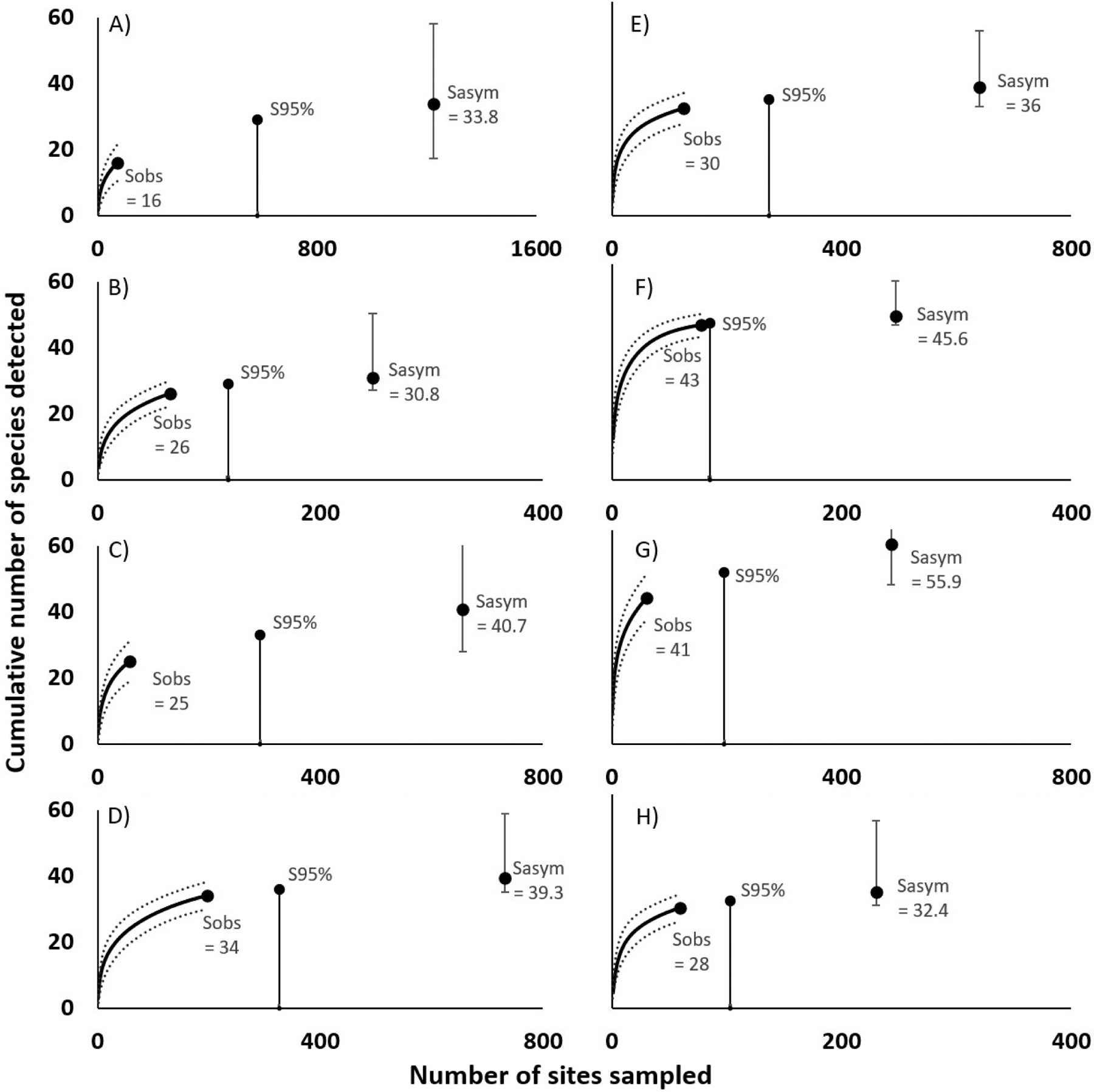
Species-effort curves for each site, showing number of observed species (Sobs) and average species-effort curve (solid line) with 95% confidence interval (CI, dashed lines). The level of effort required to sample the estimated species total (Sasym; error bars show 95% CI) and 95% (S95%) of Sasym are indicated. Milwaukee (A–C, 2017–2019; D, combined), Cleveland (E, combined data), Detroit River (F, combined data), Saginaw River (G), St Joseph River (H).

Survey performance improvements notwithstanding, a full species census was not obtained for any site (Figure 3). The number of “unique species” (species detected only one time during a survey) was greater than the number of “duplicate species” (species detected exactly two times in a survey) for all sites (except Detroit 2018), and estimated species richness is higher than observed richness in all cases (Table 2). The percentage of the estimated species pool that was detected at each site (based on the full complement of survey data for each) was 73% for Saginaw, 86% for St Joseph River, 87% for Milwaukee, 89% for Cleveland, and 97% for Detroit River. The estimated sample effort required for a comprehensive species tally (i.e. S100%) varies by site but is substantial, ranging from 169 additional samples at Detroit River to 536 samples at Milwaukee (Table 2). Estimates for the additional effort required to detect 95% of the predicted species pool (S95%) range from 7 samples at Detroit River to 149 samples at Cleveland.

### Species distribution

Species richness varied across habitats. The only habitat variables measured *in situ* were water temperature, Secchi transparency, and water depth. Only water depth was measured at all sampling locations. Maximum values for Secchi transparency varied between 1.5 m (Saginaw) and 3.6 m (Cleveland; Figure S3), while maximum depth varied between 4.2 m (Detroit River) and 11.2 m (Cleveland; Figure S4). Species richness measures decreased as depth increased. No species were detected below 8m depth and most were detected at depths less than 6m (Figure S5).

Observed and predicted richness measures as predicted from the forest classification models were significantly correlated for all models but model performance varied (ranging from a high of 0.88 to a low of 0.46) (Table 5). A model based only on water depth explained 50% or more of the variation in observed species richness for all sites except the Saginaw River. For four of the five sites, the most accurate model included all variables, but a reduced model based on just five variables (depth, fetch, percent littoral zone, and two distance measures (to boat ramps and marinas) explained 70% or more of the variation in observed richness for all sites. At the Saginaw River, model accuracy was highest when only the “top 5 variables” were considered. The three most meaningful variables in the model (i.e. with the largest importance values) were natural features, including mean depth, percent littoral area, and maximum fetch (Table 6). However, two variables representing proxies for anthropogenic points of introduction (distance to boat launch and distance to marina) were consistently among the “top 7” most important variables at all sites (out of 18 total variables considered in the model).

## Discussion

In nine surveys across five Great Lakes’ coastal sites we detected sixty-one aquatic plant species, one-fifth of which were non-native to the basin. Notable detections included *Stukenia filiformis* in Cleveland, a native species that was presumed to be extirpated from Ohio, and the first recorded observation of non-native *Cabomba* at our St Joseph River site. Although the effort was substantial at each site and resulted in detection of species like these, rarefaction suggests that a greater effort than the one undertaken would be necessary to achieve high-confidence detection of the rarest species at each site (i.e. > 95% likelihood of detection). As has been reported for fish and invertebrates in Great Lakes ports and coastal areas, we show that allocating proportionally more effort to habitats that are likely to support plants can improve survey performance (i.e. adaptive sampling; Trebitz et al. 2009; Hoffman et al. 2011; Silver et al. 2017; Harris et al. 2018). We discuss the role of survey design for achieving detection efficiencies and the sampling effort required for comprehensive detection of non-native aquatic plants.

### Adaptive sampling

For our first surveys (in 2017), a dispersed sampling approach with survey effort distributed more or less evenly across each site resulted in a low rate of detection, with nearly half (46%) of the sample units at Milwaukee and more than one-third (39%) of sample units at Cleveland absent any plants (Figure S6). The average depth of the sample units with zero plant detections was 7.0 m and 6.9 m, respectively for Milwaukee and Cleveland (versus average depth of 3.6 m and 3.5 m for sample units where plants were detected at those sites). When we modified our survey design at Milwaukee in 2018, allocating 75% of our survey effort to locations < 4 m deep, we observed a 34% reduction in the proportion of sample units with zero detections and the number of species detected increased from 16 to 26 species even though 8 fewer sample units were visited. Similar improvements in survey efficiency (relative to a dispersed sampling design) were observed at Milwaukee and Cleveland in 2019 when surveillance was constrained to areas < 6 m deep and directed proportionally to areas of high predicted species richness (based on detection patterns from 2017 surveillance). Our findings are consistent with other studies in Great Lakes coastal areas where maximum rooting depth and highest average biomass values of submerged vegetation are generally < 4 m (Angradi et al. 2013, St Louis River Estuary; Skubinna et al. 1995, Saginaw Bay; Hudon et al. 2000, St Lawrence River) and are similar to observations from other lakes where emergent and floating-leaved aquatic plants seldom grow in waters > 3 m deep (Canfield and Hoyer 1992).

The tendency for aquatic plant incidence and biomass to decrease with depth has been attributed to various factors, including temperature and hydrostatic pressure (Hutchinson 1975; Middelboe and Markager 1997), but most notably to the availability of light (Barko et al. 1986). Various models of light extinction based on scattering and absorbance by particulate matter and dissolved organics have been developed, but as a general rule of thumb, one can estimate the depth to which plants will occur (the euphotic zone) as 2–3 times the Secchi depth (French et al. 1982). For most of our sites the estimated euphotic zone (derived from Secchi depth readings at multiple locations across each site) was shallower than the maximum depth at these sites. This suggests that light availability (as a function of depth) was likely to be a limiting factor for plant growth in some portions of most sites (Table S4), and that for many sites it will be sensible to constrain survey design by depth to avoid allocating effort to deeper water habitats that cannot support plants.

However, depth may not be a limiting factor for plant establishment at all sites (e.g. shallow sites with high water clarity) and even at deeper sites it may not be the only factor influencing aquatic plant distribution. At our Detroit River site, where the maximum estimated euphotic zone was nearly twice the maximum observed depth, we chose to allocate survey effort for the second year of sampling based on patterns of species richness from the initial survey. We used a similar approach for the second-year survey at Cleveland and for the final survey at Milwaukee (both in 2019). We chose to use species richness as our proxy for plant occurrence in these surveys because richness is a function of all habitat variables and introduction pathways acting together to define whether plants will be present or absent, abundant or sparse (Barko et al. 1986; Lacoul and Freedman 2006).

We expected that targeting areas of high species richness would improve our detection rates for both native and non-native species. Indeed, native and non-native richness were positively correlated at all sites and for all surveys. This observation is noteworthy because it contradicts the prevailing paradigm of an “invasion paradox” which posits independent lines of evidence for a positive native-exotic richness relationship (NERR) at broad spatial scales and a negative NERR at fine scales (Fridley et al. 2007). Yet, a recent global meta-analysis of native and exotic richness across various habitats (terrestrial and aquatic), found no evidence of a systematically negative NERR (Peng et al. 2019) and, consistent with our findings, other observational studies of plant richness have demonstrated a positive NERR at the local scale (Stohlgren et al. 2001; Trebitz and Taylor 2007; Landi and Chiarucci 2014). Furthermore, surveys of fish and invertebrate communities in Great Lakes’ coastal areas have demonstrated that targeting species-rich habitats reduces the number of species-poor hauls and results in an overall increase in detection of native and non-native species (Trebitz et al. 2009; Hoffman et al. 2011). Taken together, these lines of evidence suggest that species richness measures can be leveraged to facilitate detection of the entire aquatic plant community, native and non-native, in Great Lakes coastal areas.

The forest classification and regression model that we used to interrogate species richness patterns in our surveys shows that just five habitat variables explain more than 70% of the observed variation in species richness for any site. Other studies that have examined the factors regulating aquatic plant occurrence in the Great Lakes have reported similar findings. At the Toronto waterfront (Lake Ontario), a model that included only fetch, water depth, and water clarity (from Secchi depth) predicted submerged aquatic vegetation (SAV) occurrence with 87% accuracy (Midwood et al. 2020). In fluvial lakes of the St Lawrence and Ottawa Rivers fetch and depth were the most important factors influencing macrophyte biomass (Hudon et al. 2000), while fetch and depth combined with bed slope and substrate size predicted occurrence of SAV with 78–86% accuracy in a Lake Superior estuary (Angradi et al. 2013). In general, focusing surveillance on shallow areas with relatively low exposure (i.e. short fetch) should increase species’ detections and improve survey performance and efficiency (compared to a more dispersed sampling design).

For some temperate lakes, natural features like fetch and slope are more important for structuring the aquatic plant community than anthropogenic drivers, including lakeshore hardening, presence of ports or marinas, and hydrologic modifications like channels and outfalls (Bertrin et al. 2017). In our study, depth, percent littoral zone, and fetch were the most important variables in the regression models but two variables associated with anthropogenic introduction pathways (distance to ramp and distance to marina) also consistently showed high importance values. Furthermore, richness measures at sample units with boat ramps (or in adjacent sample units), while variable, exceeded average richness values in several instances (Table S5). These findings are similar to results from other studies where the occurrence of invasive macrophytes in lakes is associated with variables characterizing human access and use (Compton et al. 2012; Davidson et al. 2015; Brainard et al. 2021). Given that our focus is on the detection of IAP, ensuring that surveys include all boat ramps, marinas, and any other obvious points of introduction is prudent and is likely to improve survey performance.

### Survey effort

Despite evidence for increasingly efficient surveys as we adaptively sampled, we detected less than 90% of the estimated richness at the sites that we sampled more than once (range, 60–87%). Our results indicate that a substantially larger survey effort would be required to detect the entire aquatic plant species pool at any of these locations, and thus ensure detection of rare, including potentially invasive, aquatic plants. A minimum of 169 additional samples would be required to detect all (S100%) species at Detroit River, and comprehensive detection at Cleveland and Milwaukee would require more than 500 additional samples at each site. A relevant risk management question is what level of detection is “good enough?” Even 95% detection, which is often touted as the desired survey performance metric, would require a much larger survey effort. At Milwaukee, as many as 326 samples may be required for 95% detection. This effort is within the range of effort suggested from other studies for high probability detection of fish and inverts (Hoffman et al. 2011, 2016), but amounts to several 3-day survey trips. For now, risk management decisions regarding whether to employ this method and how much effort to apply may benefit from consideration of the relative strengths and weaknesses of this approach when compared to existing alternative approaches for aquatic plant surveillance and monitoring.

Numerous states, including several Great Lakes states, have developed and implement monitoring programs for aquatic vegetation (Bartodziej and Ludlow 1997; Higman et al. 2010). Existing programs range from relatively simple protocols for qualitative inventory of species richness or plant cover by visual observation to more complex protocols, including quantitative approaches that combine visual observation with plant collection at multiple sample sites and that are used to statistically compare plant frequency and abundance over time (i.e. status and trends monitoring; Mikulyuk et al. 2010; Madsen and Wersal 2017). Although these protocols could facilitate detection of invasive aquatic plants, they are not expressly designed for early detection of the rarest plants, which is the goal of an early detection sampling protocol for Great Lakes’ coastal areas. In fact, the properties that make for a good index survey (e.g. consistency, repeatability, and routine interception of the same taxa) are in opposition to the properties that make for an efficient species detection program (i.e. variable equipment or locations to sample each taxa twice but no more than that). Conventional “status and trends” plant monitoring protocols are also generally designed for surveys on inland lakes, most of which are smaller than 200 hectares. Most are also not probabilistic, meaning that our ability to infer how likely it is that the survey detected all species present in the system is limited. Systematic sampling methods (e.g. point-intercept) could facilitate quantitative inference, but the sheer effort to systematically sample large (400+ hectares) sites may limit the utility of such a sampling design. For example, based on the systematic sampling design used by Wisconsin DNR (Mikulyuk et al. 2010), we estimate that more than 600 sample points would have to be sampled to survey the littoral area within Milwaukee harbor.

“Rapid” sampling alternatives employing rake sampling and visual observation at points of entry, or meanders through littoral areas are commonly employed for the purpose of detecting IAP (Perleberg et al. 2019; Higman et al. 2010; Michigan Department of Natural Resources, 2020). The point of entry surveys operate on the assumption that a novel species will settle or establish at a select few points of entry, but these surveys are not quantitative or probabilistic and are therefore unable to test that assumption. Visual meanders could be an especially efficient way to sample for floating and emergent aquatic plants and meander surveys have been shown to be more efficient than systematic sampling for detection of rare plants (Goff et al. 1982; Huebner 2007). However, as with point of entry surveys, the ability to estimate sampling completeness from visual meanders is limited and such surveys could fail to detect submerged species in deeper habitats or where visibility is limited.

**Figure 4.**
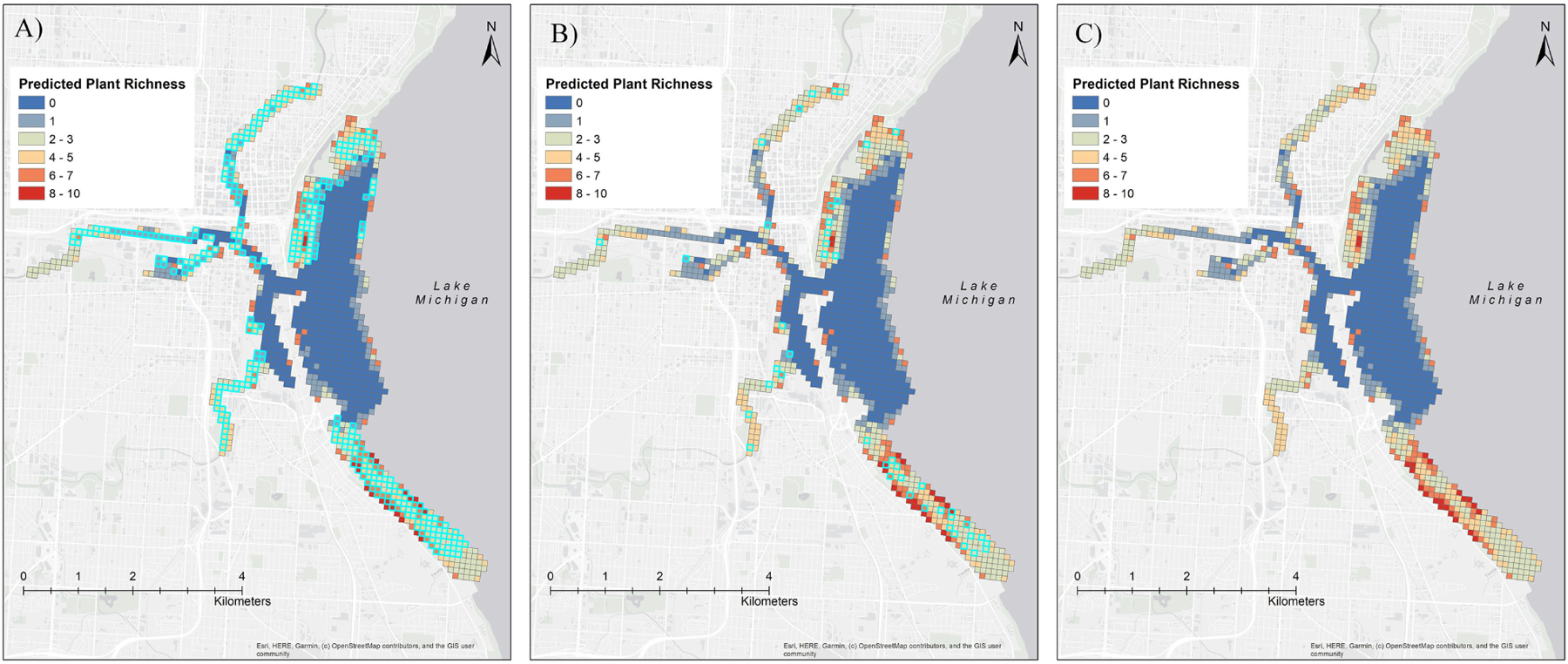
The predicted richness surface (from forest classification model) showing, (A) predicted plant richness for each grid (sample unit), (B) the grids sampled with rakes (highlighted), and (C) the cells intersected with boat track during the 2019 Milwaukee survey (highlighted).

Admittedly, the sampling approach we employed, which requires distinguishing plants to the species level to facilitate quantitative assessment of survey completeness via rarefaction, will involve significantly more effort than either point of entry or visual meander surveys and it requires a degree of taxonomic expertise that may not be necessary to detect the subset of invasive species of greatest concern. Yet, retaining the ability to measure survey performance is critical for evaluating whether or not the sampling effort has met the surveillance objective (i.e. early detection). Our approach offers a sort of “best of both worlds” scenario, in that it provides quantitative estimates of survey performance but also requires traversing across a substantial portion of the survey location, which functionally increases the potential to encounter IAP that may be present at the site. As an example, in our 2019 Milwaukee survey we actively sampled (with rakes and by visual observation) at only 6% (45 of 682) of the sample units where the forest classification and regression model predicted presence of plants. However, analysis of the boat track indicates that we passed through almost 50% (328 of 682) of the sample units with predicted richness > 0 (Figure 4). Although we did not thoroughly search these additional sample units, there was at least the potential to encounter plants that would have warranted closer inspection, especially floating or emergent growth forms or dense submerged plant beds. Notably, the survey method we describe would not be well suited for coastal wetland surveys, as inundated mud-flats or wet meadows may be best sampled via wading or other survey techniques (Trebitz and Taylor 2007; Johnston et al. 2007; Croft and Chow Fraser 2009; Uzarski et al. 2017). However, the design could easily be adapted for early detection of IAP at inland lakes by implementing a randomized sampling design for initial surveys, including visual meander and adaptive sampling to ensure communities of potential interest are not missed during the survey, assessing survey performance, and adaptively weighting survey effort towards the most suitable habitats and probable points of introduction in subsequent surveys.

## Conclusions

Our efforts, spanning five sites and three Great Lakes states, provide some helpful insights for planning and implementing surveillance efforts for early detection of non-native aquatic plants in open water habitats of Great Lakes coastal areas. Plant communities are inherently patchy and identifying the locations and associated drivers of this patchiness can improve surveys. For the sites that we surveyed, representative of sites across the basin, we improved survey performance (based on rarefaction) when we allocated proportionally more sample effort to shallow habitats (< 6 m) or species-rich areas. A forest classification regression model revealed that depth explained most variation in species richness patterns, but including fetch, percent littoral area, and distance to marinas and boat ramps increased model accuracy. Developing a richness surface from these habitat variables and then allocating survey effort proportionally towards areas of high predicted richness could be a good strategy to facilitate efficient sampling of the aquatic plant community at sites that have never been sampled. Reserving some effort for locations that might generally be assumed to be absent of plants (e.g. deeper sites, or exposed sites) is sensible to avoid the perception that new non-natives were not detected simply for lack of effort (Elzinga et al. 2009) and to minimize the risk of missing invasive species that may favor deeper habitats (Florêncio et al. 2021), but most effort should target shallow, sheltered areas and points of entry (e.g. boat ramps).

Survey locations within the broader sample frame should be selected probabilistically (e.g. using the “create spatially balanced points tool” in GIS) to facilitate quantitative assessment of survey performance, but performance goals should be realistic. For example, our results suggest that at least 100 sample units and perhaps as many as 300 sample units would need to be surveyed to detect 95% or more of the estimated richness at a site. Although the effort to detect the rarest plants is substantial, the method we describe has some advantages compared to existing alternatives (e.g. point of entry and systematic point-intercept surveys). Most importantly, this approach is quantitative and therefore enables the practitioner to evaluate survey performance and estimate what additional effort may be required for high confidence detection of the entire plant community, including potential IAP. Although survey performance metrics were derived solely from what is collected and observed within the sample units, the boat meander from sample unit to sample unit suggests that from a practical perspective these surveys cover space efficiently. Applying our approach to other Great Lakes coastal areas where IAP introduction risk is high will help to further clarify the level of effort required to adequately sample Great Lakes coastal habitats for early detection of invasive aquatic plants. Continued adaptation of this approach to improve survey performance and survey design is warranted.

## Supplementary Material

Figures 1 **Figure S1.** Scatter plots of native and non-native species richness.
**Figure S2.** Box plots of total, rare 5, rare 20 and non-native species richness.
**Figure S3.** Box plots of Secchi transparency (meters) for each survey by year.
**Figure S4.** Box plots of water depth (meters) for each survey by year.
**Figure S5.** Scatter plots of species richness versus depth (meters) for all surveys.
**Figure S6.** Frequency histograms showing the number of species detected per sample unit.

Table 1 **Table S1.** Habitat attributes used for forest classification model.
**Table S2.** Species list from aquatic plant surveys at five sites, 2017–2019.
**Table S3.** Growth form and native status of species detected for all sites combined, 2017–2019.
**Table S4.** Water depth, Secchi transparency, and estimated euphotic zone for all surveys.
**Table S5.** Observed species richness at sample units containing boat ramps.

## Figures and Tables

**Table 1.** Spatial coverage of survey effort, showing for each site and for zones within each site the number of sample units, the number of sample units surveyed (with rakes), the proportion of sample units surveyed (with rakes), and the total area.

	No. sample units	No. sample units surveyed	Proportion of sample units surveyed	Area (hectares)
**St Joseph (2017)**	**311**	**59**	**19%**	**149**
lower river	168	35	21%	80
upper river	123	22	18%	65
Paw Paw confluence	20	2	10%	4
**Saginaw River (2018)**	**865**	**60**	**7%**	**534**
shallow (< 4m)	726	52	7%	413
deep (> 4m)	139	8	6%	121
**Cleveland (2017)**	**930**	**69**	**7%**	**626**
outer harbor	605	28	5%	492
inner harbor	125	22	18%	57
river	200	19	10%	76
**Cleveland (2019)**	**906**	**56**	**6%**	**642**
90^th^ percentile: 0.6	0	0	0%	1
80^th^ percentile: 0.5	83	15	18%	55
70^th^ percentile: 0.4	63	7	11%	46
60^th^ percentile: 0.3	36	5	14%	30
50^th^ percentile: 0.2	87	4	5%	59
< 50^th^ percentile: 0.1	637	25	4%	451
**Detroit River (2018)**	**685**	**39**	**6%**	**409**
**Detroit River (2019)**	**685**	**39**	**6%**	**409**
90^th^ percentile: 0.6	42	9	21%	19
80^th^ percentile: 0.5	44	1	2%	17
70^th^ percentile: 0.4	56	10	18%	30
60^th^ percentile: 0.3	73	5	7%	42
50^th^ percentile: 0.2	61	4	7%	42
< 50^th^ percentile: 0.1	409	10	2%	260
**Milwaukee (2017)**	**1192**	**73**	**6%**	**835**
marinas	296	21	7%	206
outer harbor	441	18	4%	414
inner harbor	191	15	8%	119
river	264	19	7%	96
**Milwaukee (2018)**	**1192**	**65**	**5%**	**752**
shallow (< 4m)	643	57	9%	223
deep (> 4m)	549	8	1%	529
**Milwaukee (2019)**	**1171**	**58**	**5%**	**792**
90^th^ percentile: 0.6	27	4	15%	27
80^th^ percentile: 0.5	51	11	22%	47
70^th^ percentile: 0.4	46	5	11%	47
60^th^ percentile: 0.3	45	6	13%	51
50^th^ percentile: 0.2	57	3	5%	60
< 50^th^ percentile: 0.1	945	29	3%	560

**Table 2.** Sample size and performance measures for each site and survey year^*^.

Site	Year	No. of samples	No. of incidences	*Uniques*	*Duplicates*	*S_obs_*	*S_est_ (95% CI)*	*S95%*	*S100%*	*Lowest possible (No. samples)*
St Joseph	2017 ^a^	59	258	6	4	28	32.4 (28.8–52.5)	44	171	S87 (2)
Saginaw	2018 ^a^	60	498	11	4	41	55.9 (44.6–102.1)	136	426	S74 (2)
Cleveland	2017 ^a^	69	232	5	3	24	28.1 (24.7–49.4)	61	214	S86 (2)
	2019 ^a^	55	272	7	4	26	32.0 (27.2–56.5)	63	202	S82 (2)
Detroit	2018	39	409	3	6	34	34.4 (34.0–39.5)	7 ^b^	24	n/a
	2019 ^a^	39	480	7	4	39	45.0 (40.2–69.3)	33	143	S87 (1)
Milwaukee	2017	73	105	6	1	16	23.4 (17.3–58.0)	509	1151	S48 (2)
	2018 ^a^	65	265	7	5	26	30.8 (27.0–50.4)	52	182	S85 (2)
	2019 ^a^	58	177	8	2	25	40.7 (28.0–108.4)	234	598	S62 (2)
Cleveland	All	125	506	6	3	30	33.7 (30.6–51.9)	149	515	S84 (4)
Detroit	All	78	889	4	3	43	44.5 (43.2–55.3)	7	169	S95 (7)
Milwaukee	all ^a^	196	547	8	6	34	39.3 (35.1–58.8)	130	536	S87 (5)

* Survey data include the number of samples, number of incidences (tally of all species occurrences), “uniques” (species captured in a single sample), “duplicates” (species captured in two samples), and observed species richness (S_obs_). Performance measures include the estimated incidence-based species richness (S_est_) with associated 95% confidence interval (CI), as well as the number of samples required to sample 95% (S95%) or 100% (S100%) of S_est_ and an estimate of the lowest possible percentage of S_est_ that could be calculated for the first one or few samples, based on Chao et al. (2009).

a indicates S_est_ was calculated from the Classic Formula because of high incidence-based CV for bias corrected estimated.

b indicates the number of samples associated with 99% of S_est_.

**Table 3.** Mean values for aquatic plant richness endpoints over individual sample units (grids) within a site.

	Year	N	Richness endpoint			
			Total	Rare-5	Rare-20	Non-native
St Joseph	2017	59	4.4	0.2	1.1	1.1
Saginaw	2018	60	8.3	0.3	1.5	1.7
Cleveland	2017	69	3.4	0.3	1.0	0.6
	2019	56	4.9	0.3	1.1	0.8
Detroit	2018	39	10.5	0.1	1.4	2.7
	2019	39	12.3	0.2	1.2	2.6
Milwaukee	2017	73	1.4	0.2	0.5	0.4
	2018	65	4.1	0.3	1.0	1.1
	2019	58	3.1	0.2	1.1	0.8

**Table 4.** Simplified Morisita similarity index values and the number of shared species among designs for the various design contrasts (yr1 = Year 1, yr2 = Year 2, yr3 = Year 3).

Site	Contrast	Shared spp	Index
Cleveland	yr1 v yr2	20	0.760
Detroit	yr1 v yr2	29	0.897
Milwaukee	yr1 v yr2	14	0.503
Milwaukee	yr1 v yr3	12	0.382
Milwaukee	yr2 v yr3	21	0.739

**Table 5.** R2 values from observed vs “model predicted” species richness at each site for the full model (all variables included; see Table S1), the reduced model (see text for details), and a depth only model (based on mean depth of the sample unit).

	Train/Test	Full Model	Reduced Variables	Depth Only
St. Joseph River	Training	0.89	0.84	0.78
	Test	0.75	0.04	0.49
	Overall	0.87	0.58	0.74
Saginaw River	Training	0.90	0.85	0.68
	Test	0.17	0.34	0.14
	Overall	0.71	0.77	0.46
Cleveland	Training	0.92	0.85	0.80
	Test	0.53	0.20	0.25
	Overall	0.87	0.74	0.75
Detroit River	Training	0.92	0.78	0.67
	Test	0.55	0.29	0.20
	Overall	0.88	0.73	0.64
Milwaukee	Training	0.92	0.85	0.78
	Test	0.73	0.48	0.13
	Overall	0.84	0.79	0.68

**Table 6.** Variable importance measures from the forest classification and regression models for each site. The values in the importance column are the sum of the Gini coefficients from all the trees for each variable listed. The values in the % column are the percentage of the total sum of Gini coefficients.

*Variable*	Cleveland		Detroit		St Joseph		Saginaw		Milwaukee	
	*Importance*	*%*	*Importance*	*%*	*Importance*	*%*	*Importance*	*%*	*Importance*	*%*
Mean depth *	1991.01	24	1957.99	23	1980.39	24	1964.46	25	2040.21	24
Percent littoral zone *	1188.52	14	1153.23	14	1039.36	12	1028.3	13	1106.26	13
Maximum fetch *	767.82	9	781.71	9	797.82	9	777.45	10	721.13	9
Min. distance to soft shore	744.48	9	754.2	9	801.09	10	690.54	9	801.77	10
Min. distance to boat launch *	551.95	7	576.49	7	550.03	7	529.8	7	566.34	7
Percent soft shore	506.19	6	510.57	6	503.37	6	451.42	6	478.91	6
Min. distance to marina *	424.68	5	418.97	5	448.33	5	385.31	5	433.1	5
Mid. Distance to docked shore	403.55	5	407.54	5	444.31	5	375.56	5	451.61	5
Min. distance to lots of docks	385.58	5	420.98	5	403.46	5	338.91	4	377.99	4
Min. distance to hard shore	360.5	4	410.31	5	386.52	5	314.22	4	391.7	5
Proportion shore to water	349.66	4	347.9	4	345.3	4	277.54	4	319.93	4
Min. distance to moderate shore	304.5	4	301.98	4	298.95	4	284.16	4	299.9	4
Percent hard shore	126.35	2	123.4	1	115.61	1	116.12	1	116	1
Percent lots of docks	109	1	104.42	1	93.17	1	82.53	1	108.38	1
Min. distance any shore type	71.32	1	61.63	1	79.01	1	62.84	1	70.68	1
Percent moderate shore	58.16	1	67.18	1	53.88	1	48.87	1	67	1
Percent docks	45.2	1	62.68	1	50.56	1	39.13	1	53.95	1
Large marina presence	29.93	0	27.76	0	33.66	0	24.62	0	23.97	0

* Indicates variables used in the reduced variables model. We excluded the two shoreline type variables (‘distance to soft shore’ and ‘percent soft shore’) for the reduced variable model for practical reasons (e.g. shoreline type is difficult to ascertain from air photos and often requires detailed site knowledge from field visits and can be a subjective exercise that makes it difficult to produce consistent data). See Table S1 for variable definitions.
