# Supplementary material for: Towards a framework for invasive aquatic plant survey design in Great Lakes coastal areas
Source: Manag Biol Invasion. Author manuscript; Available in PMC 2023 Feb 4. (PMC9157784; doi:10.3391/mbi.2022.13.1.03)
Supplement: Figures 1 — Figure S1. Scatter plots of native and non-native species richness. Figure S2. Box plots of total, rare 5, rare 20 and non-native species richness. Figure S3. Box plots of Secchi transparency (meters) for each survey by year. Figure S4. Box plots of water depth (meters) for each survey by year. Figure S5. Scatter plots of species richness versus depth (meters) for all surveys. Figure S6. Frequency histograms showing the number of species detected per sample unit. [file NIHMS1786116-supplement-Figures_1.pdf]

Supplementary material

Recommended citation:

Tucker AJ, Annis G, Elgin E, Chadderton WL, Hoffman J (2022) Towards a framework for invasive aquatic plant survey design in Great Lakes coastal areas. *Management of Biological Invasions* 13(1): 45–67, https://doi.org/10.3391/mbi.2022.13.1.03

Figure S1. Scatter plots of native and non-native species richness.

Figure S2. Box plots of total, rare 5, rare 20 and non-native species richness.

Figure S3. Box plots of Secchi transparency (meters) for each survey by year.

Figure S4. Box plots of water depth (meters) for each survey by year.

Figure S5. Scatter plots of species richness versus depth (meters) for all surveys.

Figure S6. Frequency histograms showing the number of species detected per sample unit.

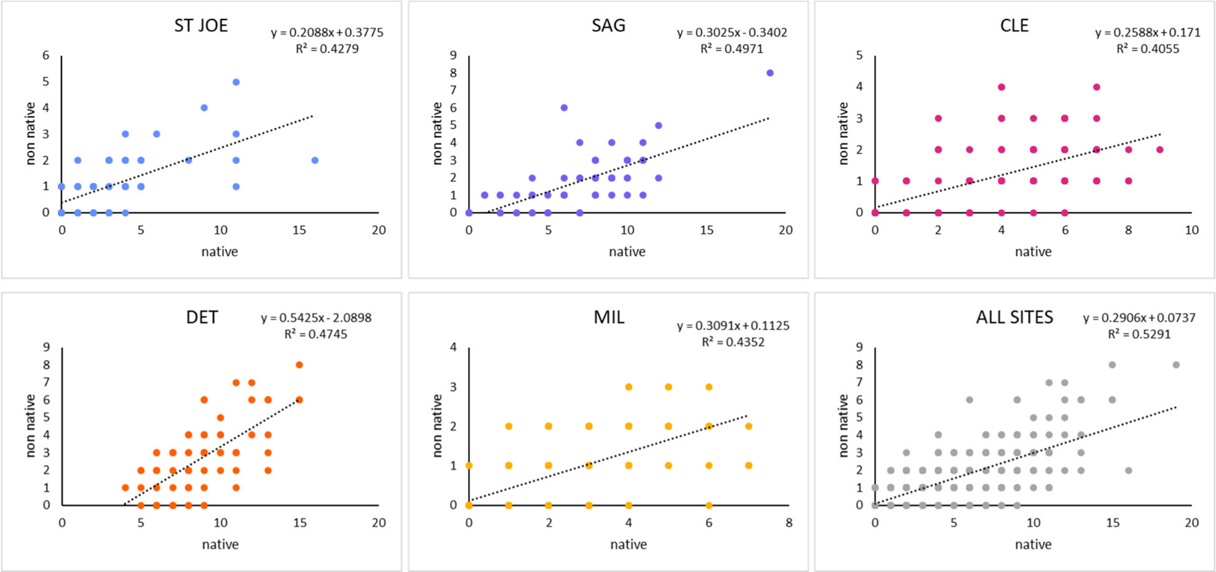

Figure S1. Scatter plots of native and non-native species richness for each site (all years) and all sites combined. Dashed lines are best fit linear regression. ST JOE = St Joseph River; SAG = Saginaw River; CLE = Cleveland; DET = Detroit River; MIL = Milwaukee

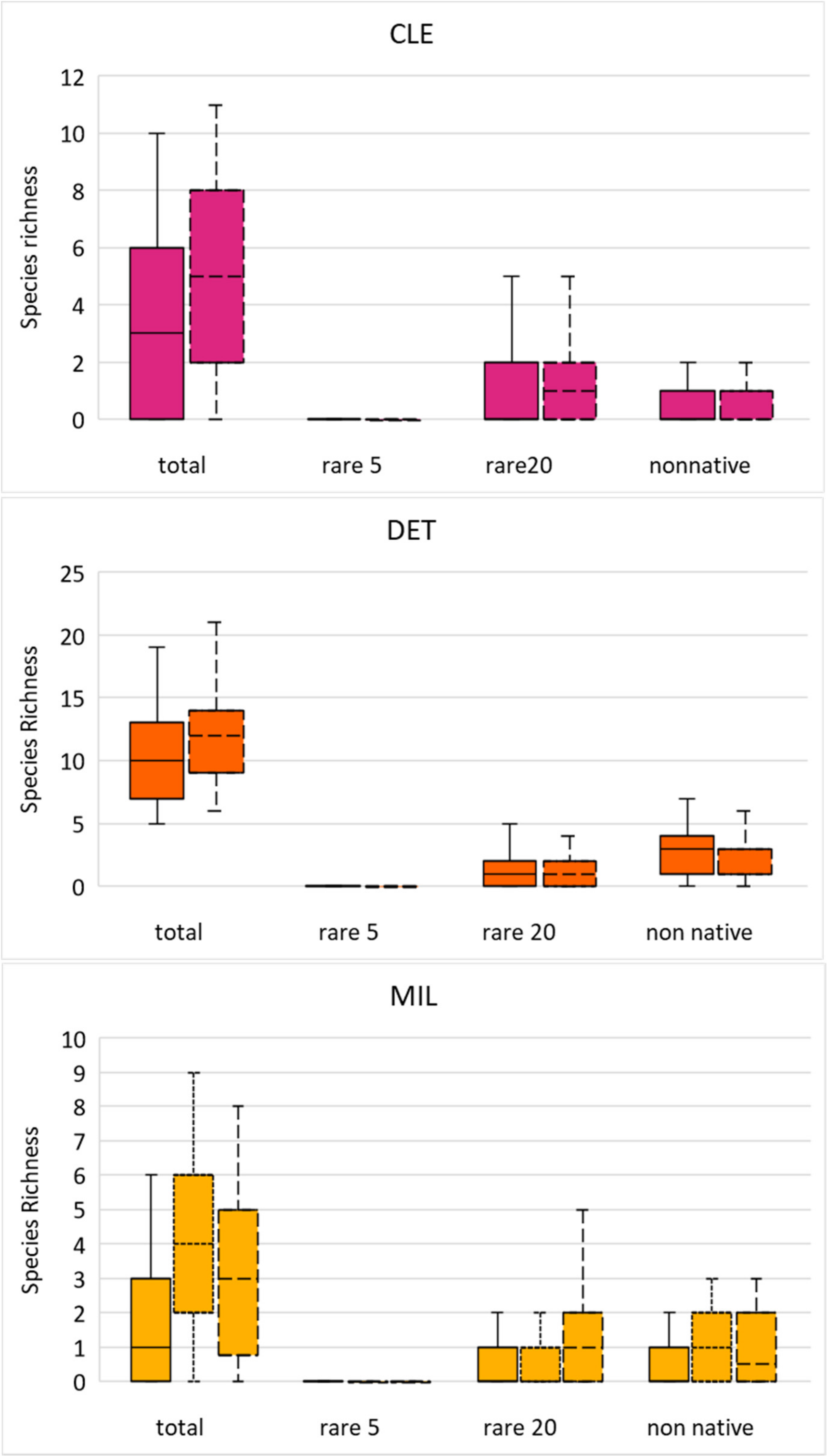

Figure S2. Box plots of total, rare 5, rare 20 and non-native species richness (as defined in text). Solid outline is year one survey. Dashed outlines are subsequent surveys. CLE = Cleveland; DET = Detroit River; MIL = Milwaukee

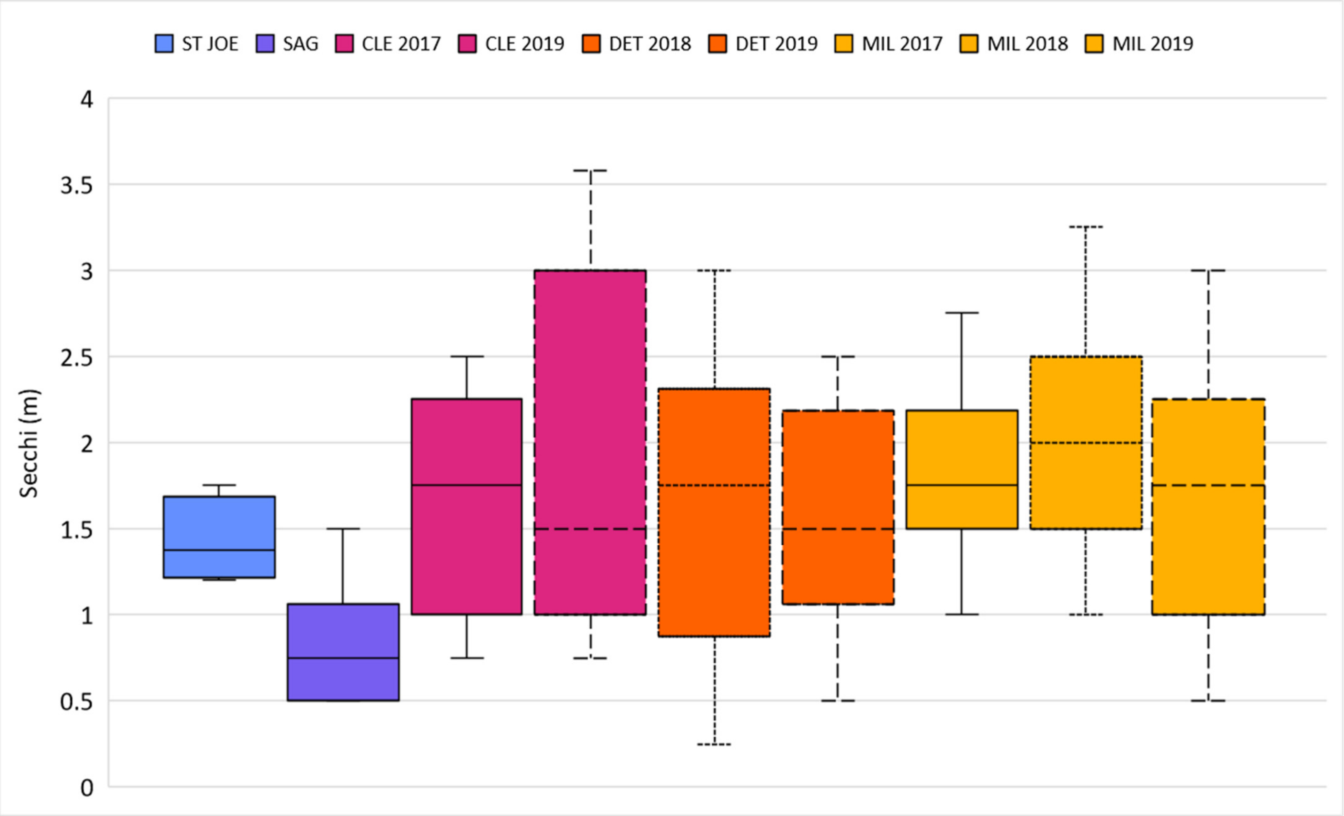

Figure S3. Box plots of Secchi transparency (meters) for each survey by year. ST JOE = St Joseph River; SAG = Saginaw River; CLE = Cleveland; DET = Detroit River; MIL = Milwaukee

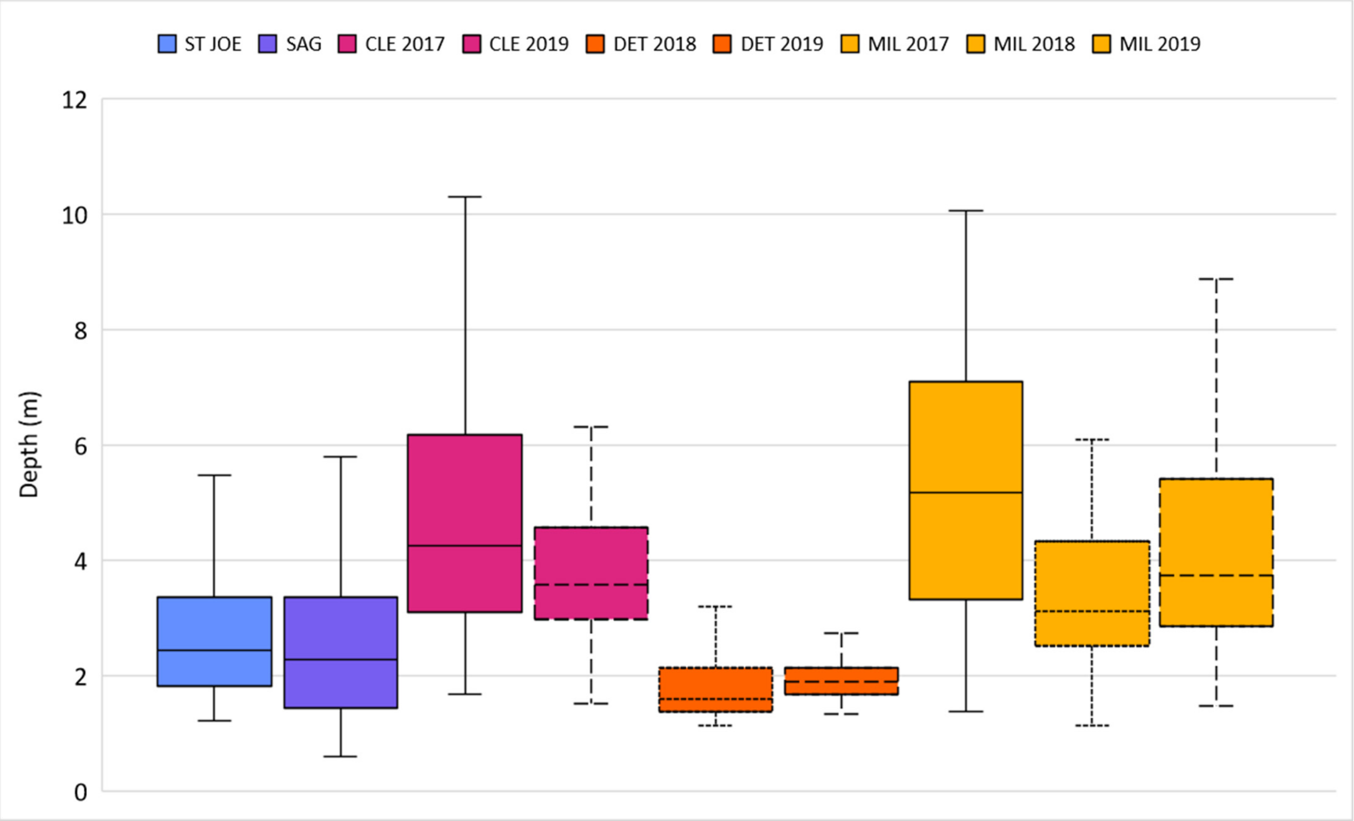

Figure S4. Box plots of water depth (meters) for each survey by year. ST JOE = St Joseph River; SAG = Saginaw River; CLE = Cleveland; DET = Detroit River; MIL = Milwaukee

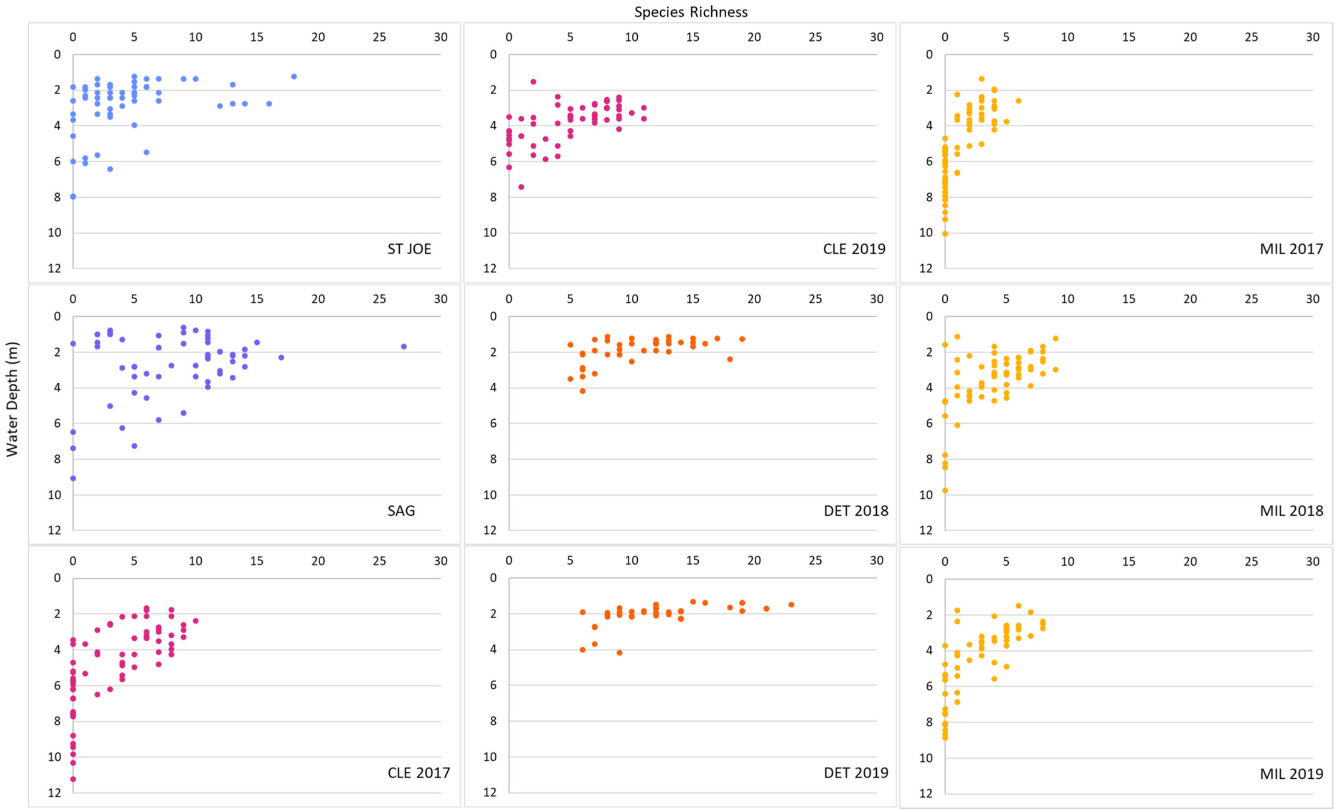

Figure S5. Scatter plots of species richness versus depth (meters) for all surveys. Dashed lines are best fit linear regression. ST JOE = St Joseph River; SAG = Saginaw River; CLE = Cleveland; DET = Detroit River; MIL = Milwaukee

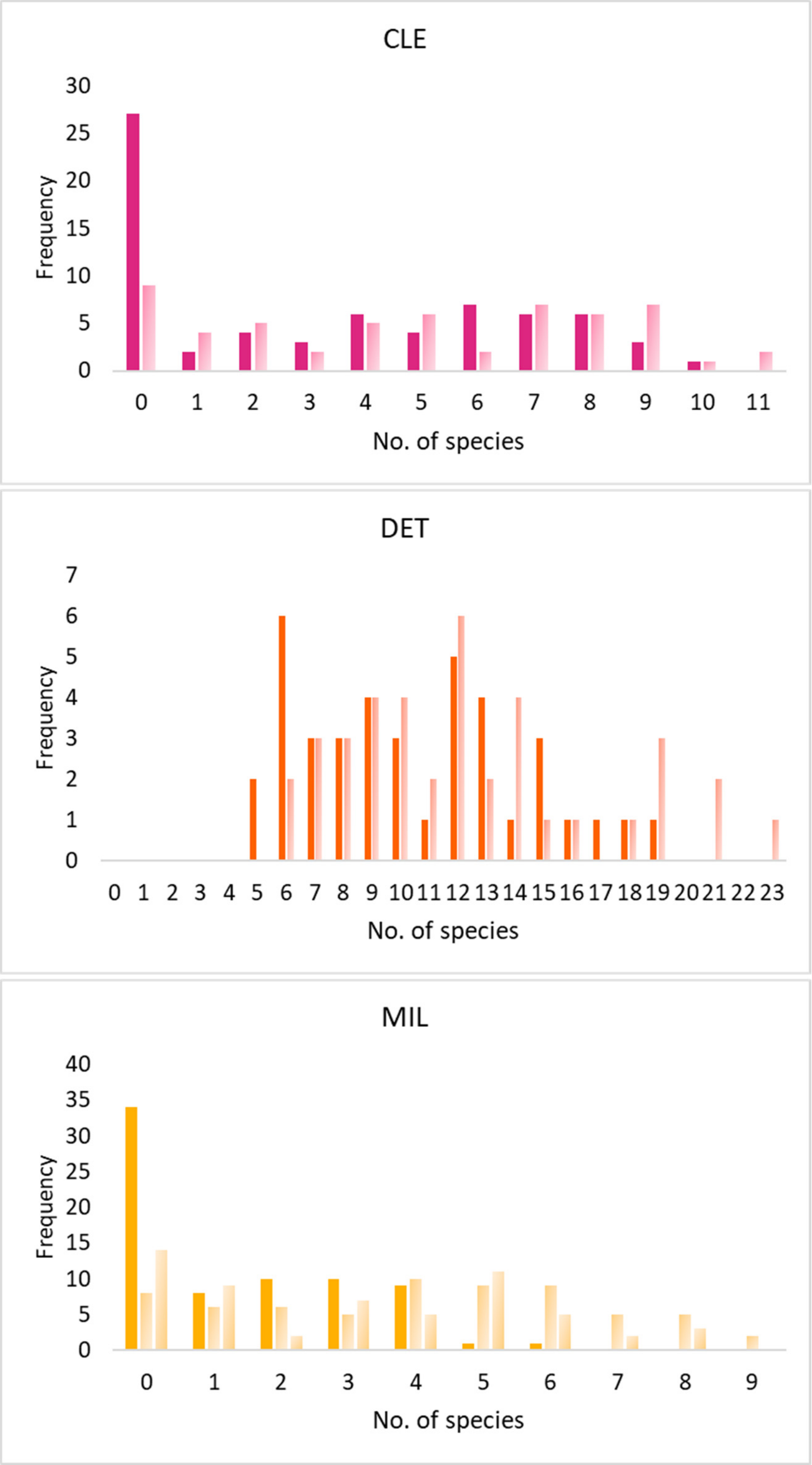

Figure S6. Frequency histograms showing the number of species detected per sample unit (grid). Dark bars are initial surveys and lighter bars are subsequent survey year(s). CLE = Cleveland; DET = Detroit River; MIL = Milwaukee
